# Determination of 2,4-Dichlorophenoxyacetic acid (2,4-D) in rat serum for pharmacokinetic studies with a simple HPLC method

**DOI:** 10.1371/journal.pone.0191149

**Published:** 2018-01-17

**Authors:** Xiao Chen, Hongling Zhang, Yanjian Wan, Xi Chen, Yuanyuan Li

**Affiliations:** 1 School of Biological and Pharmaceutical Engineering, Wuhan Polytechnic University, Wuhan, China; 2 College of Health Science and Nursing, Wuhan Polytechnic University, Wuhan, China; 3 Wuhan Center for Disease Control and Prevention, Wuhan, China; 4 Key Laboratory of Environment and Health, Ministry of Education & Ministry of Environmental Protection, Wuhan, China; 5 State Key Laboratory of Environmental Health (Incubating), School of Public Health, Tongji Medical College, Huazhong University of Science and Technology, Wuhan, China; Future University, EGYPT

## Abstract

2,4-Dichlorophenoxyacetic acid (2,4-D) is a chlorophenoxy herbicide used worldwide. We describe a high-performance liquid chromatography (HPLC) method with UV detection for the determination of 2,4-D in female and male rat serum. This allows to observe the change of serum 2,4-D concentration in rats with time and its pharmacokinetics characteristics with a simple, rapid, optimized and validated method. The serum samples are pretreated and introduced into the HPLC system. The analytes are separated in a XDB-C_18_ column with a mobile phase of acetonitrile (solvent A) and 0.02 M ammonium acetate (containing 0.1% formic acid) (solvent B) using a gradient elution at a flow rate of 1.0 mL/min. The wavelength for UV detection was set at 230 nm. Calibration curve for 2,4-D was constructed over a range of 0.1–400 mg/L. The method was successfully applied to study the pharmacokinetics of 2,4-D in rats in this study. After oral administration of 300 mg/kg and 60 mg/kg 2,4-D, the mean *C*_max_ values were 601.9 and 218.4 mg/L, the AUC_0→∞_ values were 23,722 and 4,127 mg×h/L and the clearance (Cl) were 1.10 and 0.02 L/(h×kg), respectively. The developed method was found to be specific, precise, reproducible and rapid.

## Introduction

The widely-used herbicide 2,4-Dichlorophenoxyacetic acid (2,4-D; CAS No.94-75-7) was first synthesized in 1941 [1, 2]. Ingestion, skin contact and inhalation are the three main pathways of human exposure to 2,4-D herbicides [3]. The overuse of 2,4-D by both the farmers and the manufacturers in the tropics has greatly damaged the health of the local ecosystem because it is deleterious to both terrestrial and aquatic living organisms [4].

The subchronic exposure of 2,4-D has shown toxic effects on the kidneys (increased kidney weight, histopathological lesions) and on the liver (increased liver weight, increased liver enzymes) [5]. Chronic exposure in rats is manifested by decreased body-weight gain, altered organ weights and hematological parameters and other biochemical changes [6]. A previous study also reported that 2,4-D could induce deleterious pathological effects on the vital organs including preneoplastic changes in the liver of Sprague-Dawley rats [7].

High-performance liquid chromatography (HPLC) is general and popular for the analysis of drug and pesticide in biological samples [8, 9]. Some HPLC methods have been established for the determination of 2,4-D residues in vegetables, fruits [10] and environmental samples [11, 12], but only a few studies have been carried out for biological samples [13, 14]. There is only limited data with respect to 2,4-D pharmacokinetic properties [15]. According to Chinese Standard Bureau (GB) 15670 “Toxicological Test Methods of Pesticides for Registration”, rat species share many of the pharmacokinetics properties of 2,4-D with humans. The rat is regarded as one of the best animals among small laboratory animals for studying the pharmaco-toxicological aspects of herbicides.

The safety evaluation of pesticide is a continuous process. As one of the most widely used herbicides in the world, 2,4-D continues to be one of the most studied pesticides, both in animals and in humans [16–18]. China is a great agricultural country and 2,4-D is used extensively because of its efficacy [19, 20]. However, the toxic effects of 2,4-D were rarely reported in the current literature in China. The most frequent method measuring the 2,4-D is by LC-MS, which is expensive and is not in general use in most of grass-roots units in China. Therefore, we developed a method using HPLC that is virtually 4–5 times cheaper than LC-MS for the determination of 2,4-D in serum and studied its pharmacokinetic characteristics in healthy male and female Sprague Dawley rats. The findings of this investigation will provide the foundation for further studies of toxicity and mechanism. It is also a scientific basis for the development of hygiene standards and countermeasures to prevent the harm of 2,4-D in the environment.

## Materials and methods

### Chemicals and reagents

2,4-Dichlorophenoxyacetic acid (2,4-D) (> 96% purity) was obtained from the Nanjing Chang Feng Agrochemical Co., Ltd. (Nanjing, China). Chromatographic grade acetonitrile and formic acid were purchased from TEDIA Company, Inc., OH Fairfield, USA. Ammonium acetate (Tiancheng Chemical Co., Ltd., Shanghai, China). HPLC-grade water was prepared using a Milli-Q purification system (Millipore, Bedford, MA, USA). All other reagents used in this study were of analytical or HPLC grade.

### Preparation of calibration standards and quality control samples

The stock solution was prepared in acetonitrile to yield a final concentrations of 1.0 mg/mL 2,4-D. The standard solutions were prepared by diluting the stock solution with acetonitrile to final concentrations ranging from 0.2 to 800 mg/L. The above solutions were stored at 4°C and brought to room temperature before use.

The standard solutions (200 μL) were added to rat serum (200 μL) to yield the calibration standards of 0.1, 1, 10, 20, 50, 100, 200 and 400 mg/L. The subsequent procedure was the same as sample preparation. Quality control (QC) samples were prepared at final concentrations of 5 μg/L (low quality control sample, LQC), 50 μg/L (medium quality control sample, MQC) and 100 μg/L (high quality control sample, HQC) with the same method used for the calibration standards. The calibration standards and QC samples were kept at -20°C until use.

### Samples preparation

An aliquot of 100 μL rat serum and 3 times volume of acetonitrile were added in a 1.5 mL Eppendorf tube and vortex-mixed for 3 min, then centrifuged at 12000 rpm for 10 min at 4°C. The supernatant was passed through a nylon membrane (0.22 μm) filter, followed by transferred into a LC vial for the HPLC analysis.

### HPLC

The HPLC system used was an Agilent 1260 infinity HPLC system with Agilent 1200 VWD (Aglient Technologies, Palo Alto, CA, USA). The chromatographic separation of the compounds was achieved using a XDB-C_18_ (4.6 mm I.D.× 250 mm, 5 μm, Agilent Eclipse, Santa Clara, CA, US) at 40°C. The mobile phase was a mixture of (A) acetonitrile and (B) 0.02 M ammonium acetate with 0.1% (v/v) formic acid. The programmed gradient was 0 min, 70% B; 5 min, 70% B; 10 min, 40% B; 12 min, 10% B; 16 min, 7% B; 10 min, 95% B; 10.1 min, 1% B; 12 min, 1% B. The column was maintained at 40°C. Sample injection volume was 5 μL. A 10 μL sample solution was injected onto the column with a flow rate of 1.0 mL/min. The wavelength of the UV detector was set at 230 nm.

### Method validation

The specificity of the assay was evaluated by comparing chromatograms of the blank, standard-spiked serum samples and single dose administration rat serum samples. The extraction recovery of the analyte was determined by comparing the peak areas of the 2,4-D from the prepared serum QC samples. The peak areas of extracted LQC, MQC and HQC were compared to the absolute peak area of the unextracted samples containing the same concentration of 2,4-D as 100%. The extraction recovery of 2,4-D was determined using six replicates of each QC samples. The calibration curves were constructed and fit by linear least-squares regression analysis. The precision of the method was evaluated by repeated analyses of QC samples (n = 3) on three consecutive days.

### Application to a pharmacokinetic study in rats

Sprague-Dawley female rats (aged 6–8 weeks; weight, 270 ± 37.3 g), male rats (aged 6–8 weeks; weight, 286 ± 42.6g) were purchased from Tongji Medical College, Huazhong University of Science and Technology Laboratory Animal Center and housed at a temperature (23 ± 3°C) in moisture-controlled (55 ± 15% relative humidity) room (specific pathogen free). The room had a controlled 12 h light-dark cycle and access to food and water *ad libitum*. The rats were divided into two groups of 8 each (female and male rats each half), and given the 2,4-D by gavage. The dose to A group rats was 300 mg/kg body weight (bw) and B group rats was 60 mg/kg body weight (bw). Oral dosing solutions were prepared in 50% dimethyl sulfoxide (DMSO). Serial blood samples (0.3 mL) were collected at the following intervals 5, 15, 30 min, 1, 2, 4, 8, 24, 48, 72 and 168 h after dosing, and were stored at -20°C until their analysis. The rat serum samples (100 μL) were processed as described above. All animal procedures were approved by the Institutional Animal Care and Use Committee of Huazhong University of Science and Technology.

### Statistics analysis

All the statistical analysis was conducted using Phoenix WinNonlin Enterprise Program v5.3. The graphs and tables were created with Microsoft Excel 2010. The maximum serum concentration (*C*_max_) and the time to reach *C*_max_ (*T*_max_) were directly obtained from the study data. *K*_a_ is the absorption rate constant. The elimination half-life (*t*_1/2_) was calculated as 0.693/*K*_*e*_, where *K*_e_ is the elimination rate constant calculated from the terminal linear portion of the log serum concentration-time curve. The area under the serum concentration-time curve (AUC) from time zero to the last quantifiable time point (AUC_0→t_) and from time zero to infinity (AUC_0→∞_) were estimated using the log-linear trapezoidal rule. Absorption half-time (*t*_1/2_*K*_a_), end elimination half-life (*t*_1/2_ *z*), volume of distribution based on the terminal phase (*V*_d_) and total body clearance (Cl) and are directly obtained from the study data processed.

## Results

### Analytical method validation

#### Specificity

**Fig 1:**
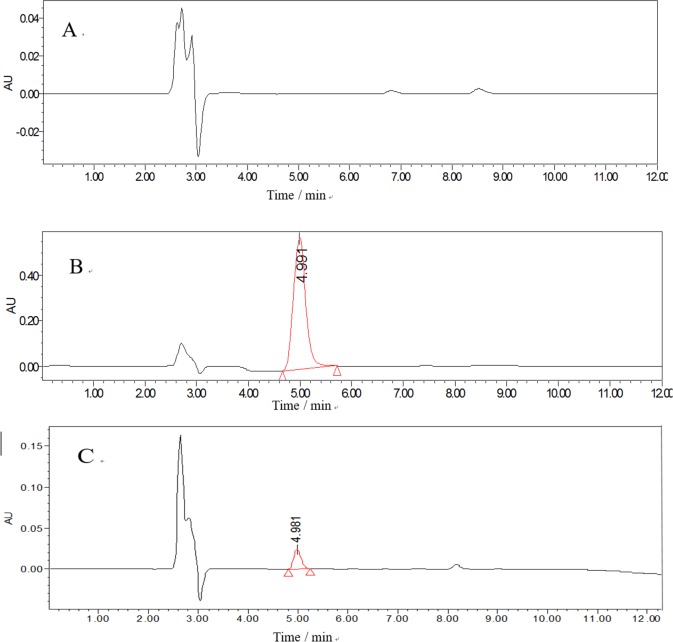
Chromatogram of 2,4-D obtained during analysis of rat serum samples. Representative chromatograms of (A) blank rat serum, (B) serum spiked with 2,4-D, and (C) serum sample obtained 15 min after oral administration 2,4-D.

Representative chromatograms for blank rat serum and blank rat serum spiked with 2,4-D are shown in Fig 1A and 1B (Fig 1). Fig 1C shows the chromatogram of the rat serum sample obtained at 15 min after oral administration of 2,4-D, and it was well resolved at retention times of (4.7 ± 0.3) min. No endogenous interference was found at the retention times of 2,4-D, indicating that the developed method is specific for 2,4-D.

#### Extraction recovery

The recovery of 2,4-D from the serum was determined in accordance with the method described in the previous section. The recoveries (mean) of 2,4-D from serum were found to be 101.9% at 5 μg/mL, 100.4% at 50 μg/mL and 100.8% at 100 μg/mL.

#### Linearity

The calibration curves were obtained by determining the peak area ratios vs. concentration of prepared calibration standards and fitted to the equation Y = 24104X -159.27. The linearity of the calibration curve was evaluated by calculating the R (regression coefficient) values. The standard curves of 2,4-D in rat serum were linear over the concentration ranges of 0.1–400 mg/L and regression coefficients (R^2^) were over 0.999 from each standard curve of seven separate runs. The lower limit of quantification (LLOQ) was defined as the lowest concentration in rat serum was 0.3 mg/L when the signal to noise ratio is 10:1 (S/N = 10).

#### Precision

Intra- and inter-day precision (as RSD) for 2,4-D in rat serum were assayed with the QC samples with known amount of 2,4-D according to the procedure described in the previous section. Results are shown in Table 1. The impression of this bioanalytical method for inter- and intra-run ranged from 0.48% to 1.90% and 0.35% to 7.26% respectively.

**Table 1:** Inter- and intra-day precision for determination of 2,4-D in rat serum.

QC samples	Nominal concentration (μg/mL)	n	Calculated concentration (μg/mL)	Precision (%RSD)
Inter day				
LQC	5.00	6	5.14 ± 0.48	3.28
MQC	50.00	6	49.3 ± 0.76	1.53
HQC	100.00	6	95.2 ± 1.90	2.00
Intra day				
LQC	5.00	6	5.05 ± 0.35	6.86
MQC	50.00	6	52.2 ± 3.93	7.54
HQC	100.00	6	102.2 ± 7.26	7.10

### Pharmacokinetic application

**Fig 2:**
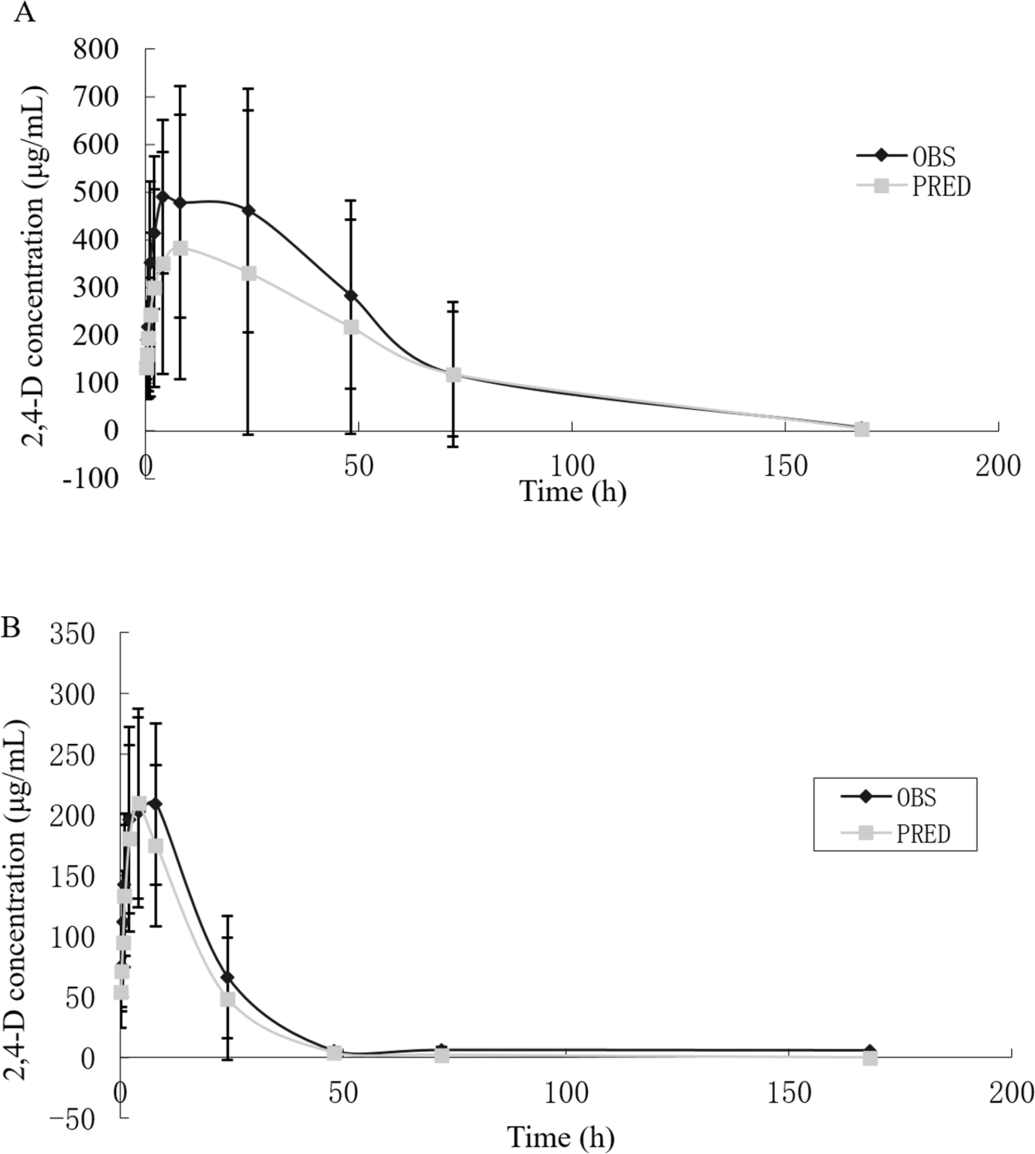
Concentration-time curve of 96% 2,4-D in rat serum. Mean serum concentration-time profile of 96% 2,4-D after oral administration of 300 mg/kg bw (A) and 60 mg/kg bw (B) to rats (n = 16). Values at 168 h were below the limit of quantification. (OBS means observed value; PRED means predicted value).

The mean values of serum concentration-time for 2,4-D following oral administration were shown in Table 2. With the LLOQ of 0.3 μg/mL, serum concentrations of 2,4-D were successfully quantified for up to 72 h after oral administration of 2,4-D to rats. The serum concentrations of 2,4-D in female rats of B group taken at 168 h were below the LLOQ. For the A group (300mg/kg), the highest mean values of 2,4-D concentrations in female and male rats were 612.3 mg/L and 520.1 mg/L, respectively, taken at 8 h. For the B group (60 mg/kg), the highest mean values of 2,4-D concentrations (223.8 mg/L) for female rats were taken at 2 h, while the highest mean values of 2,4-D concentrations (198.9 mg/L) for male rats were taken at 4 h. 2,4-D was not detected at 168 h for female rats in the B group. The difference between female and male rats happened at 5 min, 2, 72 h for the A group and 5, 15 min, 1, 72 h for the B group. The differences are the greatest at 72 h at high dose (almost 10times). The sex differences were not always statistically significant. 2,4-D was more rapidly eliminated from serum by male rats than by female rats. The basic pharmacokinetic parameters of 2,4-D in rats were calculated based on the serum concentration data. After oral administration of the 2,4-D (300 mg/kg body weight) and after oral administration of the 2,4-D (60 mg/kg body weight), the parameters were for *t*_1/2_, respectively 16.6 h, and 6.84 h, for *t*_1/2_*K*a, 4.14 h and 2.44 h, for *t*_1/2_*z*, 90.8 h and 15.1 h, for *T*_max_, 17.5 h and 5.25 h, for *C*_max_, 601.9 mg/L and 218.4 mg/L, for *V*_d_/F, 0.68 L/kg and 0.16 L/kg, for *Cl*/F, 0.10 L/h/kg and 0.02 L/h/kg, and for AUC_0→t_, 20.726 mg/L×h and 4,105 mg/L×h. These are summarized in Table 3. According to the principle of Akaike Information Criterion (AIC) and the value of fitting degree (R^2^), we judged the kinetics of 2,4-D in rats to be conform to one-compartment model, weight coefficient is 1/c^2^, the serum concentration results after fitting as shown in Fig 2.

**Table 2:** Mean serum concentration (mg/L) of 2,4-D with time, after oral administration of 300 mg/kg and 60 mg/kg to female and male rats.

	A group (300 mg/kg)		B group (60 mg/kg)	
Time	Female	Male	Female	Male
5 min	184.1 ± 38.1	104.0 ± 34.7*	71.8 ± 11.1	37.6 ± 19.0*
15 min	239.0 ± 83.5	149.3 ± 33.7	108.9 ± 25.1	44.1 ± 6.2*
30 min	277.0 ± 165.5	168.8 ± 19.3	147.1 ± 38.9	81.2 ± 38.3
1 h	458.8 ± 103.9	217.6 ± 45.1	195.5 ± 44.2	95.9 ± 41.6*
2 h	545.1 ± 107.7	303.1 ±73.1*	223.8 ± 65.9	175.9 ± 47.8
4 h	501.9 ± 100.9	389.9 ± 115.6	213.9 ± 49.1	198.9 ± 66.6
8 h	612.3 ± 97.6	520.1 ± 113.6	203.3 ± 73.4	183.1 ± 54.2
24 h	453.3 ± 120.9	280.0 ± 174.7	73.4 ± 66.9	57.2 ± 46.6
48 h	423.6 ± 101.4	157.7 ± 159.1	8.0 ± 1.2	9.8 ± 6. 5
72 h	67.2 ± 37.4	6.2 ± 0.8**	4.9 ± 0.1	6.1 ± 0.4**
168 h	5.0 ± 0.3	6.2 ± 1.3	Not Detected	5.5 ± 0.2

Significant difference between sexes at same dose and time point by a Student’s *t*-test (* indicates *p* < 0.05, ** indicates *p* < 0.01)

**Table 3:** Pharmacokinetic parameters of 2,4-D after oral administration of 300 mg/kg bw and 60 mg/kg bw to rats (n = 16 for each group).

Parameters	Oral administration doses	
	300 (mg/kg bw)	60 (mg/kg bw)
*t*_1/2_ (h)	16.6 ± 13.9	6.84 ± 3.48
*t*_1/2_*K*_a_ (h)	4.14 ± 5.10	2.44 ± 1.39
*t*_1/2_*z* (h)	90.8 ± 170.2	15.1 ± 11.1
*T*_max_ (h)	17.5 ± 9.06	5.25 ± 2.38
*C*_max_ (mg/L)	601.9 ± 142.9	218.4 ± 73.2
*K*_a_ (1/h)	6.60 ± 12.28	0.38 ± 0.25
*K*_e_ (1/h)	0.09 ± 0.09	0.14 ± 0.08
*V*_d_/*F* (L/kg)	0.68 ± 0.63	0.16 ± 0.12
Cl/*F* (L/h/kg)	0.10 ± 0.16	0.02 ± 0.01
AUC_0→t_ (mg/L)	20726 ± 20503	4105 ± 1979
AUC_0→∞_ (mg/L×h)	23722 ± 22609	4127 ± 2017

*t*_1/2:_ the elimination half-life *K*_e_: the elimination rate.

*t*_1/2_*K*_a_: absorption half-time C1: total body clearance

*t*_1/2_ *z*: end elimination half-life *V*_d_: the volume of distribution based on the terminal phase

*C*_max_: the maximum serum concentration. AUC: the area under the serum concentration-time curve

*T*_max_: the time to reach *C*_max_ AUC_0→t_: AUC from time 0 to the last quantifiable time point

*K*_a_: the absorption rate constant AUC_0→∞_: AUC from time 0 to infinity

## Discussion

The 2,4-D has been the subject investigation for many years [21–23]. Only a few studies have been carried out in biological samples, such as canine plasma [13]. Chinese Bureau of Standards GB 15670 “Toxicological Test Methods of Pesticides for Registration” requires that at least two dose levels need to be selected in a single infection study: no observed adverse effect level (NOAEL) should be observed in the low dose level however toxic effects or toxic kinetics parameters changes should be observed in the high dose level. The dosage level should not cause a high mortality rate which may impact on the evaluation of the experimental results. Based on the lethal dose 50 (LD50) and our preliminary experimental results, we adopted 300 mg/kg bw and 60 mg/kg bw as the oral administration high dose and low dose respectively.

This method allows a determination of 2,4-D in rat serum by using a gradient elution for better resolution and peak shape. The retention time is 4.7 min in our study, while 10.5 min and 14.9 min were taken respectively in the previous studies [13, 14]. Although the retention time of 2,4-D by gas chromatography [24] 3.30 min is shorter than ours, the pretreatment it requires is much more elaborate than the present method. The acetonitrile we used for solvent in the experiment is effective, general and of minimal toxicity for people in the laboratory. Intraday and interday precision values of the previous HPLC method in canine plasma [13] were <10.5% and <11.8%, respectively, whereas that of the present method were < 2.27% and < 7.17%. The mean coefficients of determination for equations was greater than 0.992 in the previous HPLC method studies, that of the present study was greater than 0.999.

In the pharmacokinetic results using the developed method, orally administered 2,4-D disappears from the serum according to a one-compartment model. There was slight difference between female and male rats, which is consistent with Robert’s findings [14]. The difference in elimination may neither be owing to differences in serum protein binding of 2,4-D nor to differences in absorption according to Griffin 's research [25]. In this study, we have tested a single oral dose of 300 mg/kg bw and 60 mg/kg bw of 2,4-D in rats and calculated the oral apparent volume of distribution of 2,4-D, which were 0.68 ± 0.63 and 0.16 ± 0.12 L/kg, respectively. These low values reveal that 2,4-D may have a narrow distribution in rats and that it is mainly distributed in the serum. This method has advantages of good stability, great specificity, celerity and low cost.

## Conclusion

In this study, we have described an optimized and valid bioanalytical HPLC-UV method for the determination of 2,4-D in rat serum. The method is sensitive, selective and reproducible. This method was successfully applied to determine 2,4-D in rat serum for pharmacokinetic studies. In this article, the pharmacokinetic parameters such as clearance rate, volume distribution of 2,4-D for oral administration in female and male rats were reported for the first time. The developed method can provide the foundation for further study of toxicity and mechanism, and a scientific basis for the development of hygiene standards and countermeasures to prevent the harm caused by 2,4-D.

## Supporting information

S1 Checklist Completed ‘‘The ARRIVE Guidelines Checklist” for reporting animal data in this manuscript (PDF). (PDF)

S1 Dataset Dataset for all serum concentration of 2,4-D with time, after oral administration of rats (XLS). (XLS)
